# *De novo* yeast genome assemblies from MinION, PacBio and MiSeq platforms

**DOI:** 10.1038/s41598-017-03996-z

**Published:** 2017-06-21

**Authors:** Francesca Giordano, Louise Aigrain, Michael A Quail, Paul Coupland, James K Bonfield, Robert M Davies, German Tischler, David K Jackson, Thomas M Keane, Jing Li, Jia-Xing Yue, Gianni Liti, Richard Durbin, Zemin Ning

**Affiliations:** 1 The Wellcome Trust Sanger Institute, Wellcome Trust Genome Campus, Hinxton, Cambridge UK; 2 Cancer Research UK Cambridge Institute, Li Ka Shing Centre, University of Cambridge, Cambridge, CB2 0RE UK; 3 Max Planck Institute of Molecular Cell Biology and Genetics, Pfotenhauerstraße 108, 01037 Dresden, Germany; 4 Université Côte d’Azur, CNRS, INSERM, IRCAN, Nice, France

## Abstract

Long-read sequencing technologies such as Pacific Biosciences and Oxford Nanopore MinION are capable of producing long sequencing reads with average fragment lengths of over 10,000 base-pairs and maximum lengths reaching 100,000 base- pairs. Compared with short reads, the assemblies obtained from long-read sequencing platforms have much higher contig continuity and genome completeness as long fragments are able to extend paths into problematic or repetitive regions. Many successful assembly applications of the Pacific Biosciences technology have been reported ranging from small bacterial genomes to large plant and animal genomes. Recently, genome assemblies using Oxford Nanopore MinION data have attracted much attention due to the portability and low cost of this novel sequencing instrument. In this paper, we re-sequenced a well characterized genome, the *Saccharomyces cerevisiae* S288C strain using three different platforms: MinION, PacBio and MiSeq. We present a comprehensive metric comparison of assemblies generated by various pipelines and discuss how the platform associated data characteristics affect the assembly quality. With a given read depth of 31X, the assemblies from both Pacific Biosciences and Oxford Nanopore MinION show excellent continuity and completeness for the 16 nuclear chromosomes, but not for the mitochondrial genome, whose reconstruction still represents a significant challenge.

## Introduction

The advent of next generation sequencing technologies (NGS) has marked the start of a new era in genomics research. Compared to the previous Sanger technology^1^, NGS has significantly lowered the cost of sequencing using massively parallel sequencing methods^2, 3^. In a typical NGS run, DNA molecules are sheared into small fragments and then clonally amplified before being sequenced. After DNA amplification, multiple fragments of the sequences obtained may cover the same genome region, so that computational algorithms can be used to concatenate and assemble such reads like a jigsaw puzzle and generate a consensus to correct for the occasional sequencing errors. The typical length of the DNA fragments sequenced is between 50 and 400 bases long^2^, and as a result, the assembly obtained from such short reads is fragmented in contigs much smaller than the actual chromosome sizes. In particular, short reads are not able to solve complex genome features like repeated regions (repeats) longer than the fragment length or copy number variations, with the typical outcome that (almost-) identical repeats are collapsed into a single element in the assembly. To overcome the high fragmentation of NGS-based assemblies and to help resolve long repeats, long-read sequencing technologies have been developed and recently adopted by the genomics community. The main characteristic of these new platforms is to work with long DNA molecules and provide reads with lengths up to hundreds of kilobases (kb). Reads of such length can be exploited in various ways. Particularly in the genome assembly field they can be used for *de novo* assembly with long-read data only, or for scaffolding of NGS-based assemblies by bridging gaps between contigs or spanning long repeats thus resolving them. A major drawback of long-read technologies is the higher rate of sequencing errors (5–20%) compared to NGS data (<1%)^2^. Such an error profile could negatively affect the assembly accuracy, but because the errors are mostly randomly distributed the majority of long-read assemblers adopt the strategy of correcting base errors algorithmically before attempting to assemble the reads.

The most established long-read technology is from Pacific Biosciences (PacBio), which uses a sequencing by synthesis approach where phospholinked nucleotides are used to synthesize the complement strand of a single stranded DNA template. For the PacBio RS II machine, commercialized since 2011, the reads are characterized by lengths in the 5–60 kb range, with an average length around 12 kb^4^. The error rate for raw reads is about 13%^4^ with errors, mostly indels, randomly distributed. After a base error correction step the reads can reach an accuracy of 99.9% and higher across the read length. Throughput can reach up to about 1 Gigabases (Gb) per run^4^. Presently, the cost of sequencing a large genome with the PacBio technology is still quite high, as it involves a high initial cost for the platform (about $700k) and about $300 per Gigabases^4^. But PacBio has recently released a new platform, Sequel, that promises to increase the throughput to up to 10 Gb per run, maintaining a long average read length and decreasing the cost per Gb sequenced.

More recently, since 2014, the Oxford Nanopore Technology company (ONT) started distributing a new innovative sequencer, the MinION. The MinION is the first handheld sequencer and measures only 2 cm × 4 cm × 9 cm and weighs about 100 g. Within a MinION flowcell, single-stranded DNA molecules are guided across a membrane through protein-based nanopores. The membrane is immersed in a saline solution with a fixed voltage across it, so that an ionic current constantly passes through the pores. The DNA-strand motion through a pore causes a variation of the current, which is constantly monitored. A basecaller then determines the sequence of bases through the pore according to the observed variation in ionic current. To date, various basecallers are available: from an older Hidden-Markov-Model based (HMM) one to more recent ones based on Recurrent Neural Networks (f.i. Nanonet https://github.com/nanoporetech/nanonet). For the data analysed in this study, we used the HMM-based basecaller, which was the first made available by Oxford Nanopore. This basecalling package infers the sequence of successive *k*-mer words (5-mer or 6-mer) that passed through a particular pore by analyzing the current variations in time. According to their estimated qualities, the reads are collected into a ‘Fail’ or a ‘Pass’ directory, the latter only including the highest quality bi-directional (2D) reads, for which both strands (template and complement) of a DNA molecule have been sequenced and then merged in a single read. Like PacBio, the MinION sequencing errors are mostly randomly distributed. They can be corrected given enough read depth, with the exception of long sequences of the same base (homomers), that are often collapsed into shorter sequences by the basecaller. Nanopore sequencing is still in its rapid development stage, and since the data produced in this study many progresses have been made regarding error rate and throughput stability. Because of its portable size, low price ($1000 initial investment for the MinION, and <$300/Gb for flowcells bought in bundles), low computing requirements and relatively low wet-lab work load, the MinION has the potential to revolutionize the genomic discipline, in particular with regard to field and clinical sequencing. A number of preliminary attempts have already been reported, ranging from *in situ* outbreak analysis and control strategy^5–7^, field DNA collection and sequencing^8^, exploration of metagenomics for clinical use^9–11^, and many more (see https://nanoporetech.com/publications). Moreover, higher-throughput machines that incorporate 5 (GridION, https://nanoporetech.com/products/gridion) or 48 (PromethION, https://nanoporetech.com/products/promethion) flowcells are in early access stage, and they are foreseen to deliver throughput similar to that of Illumina HiSeq per run.

In this paper, we report a comparative study on the yeast genome assemblies from three different sequencing platforms: MiSeq from Illumina (NGS), and the long-read platforms PacBio RS II and ONT MinION. Apart from the reference yeast strain, *Saccharomyces cerevisiae* S288C, we also sequenced and assembled three other yeast strains, SK1, and the two *Saccharomyces paradoxus* N44 and CBS432. We explored the results in terms of accuracy, time and memory consumption of a variety of existing pipelines for long-reads-only *de novo* assembly, and for scaffolding of MiSeq-based assemblies using long reads. For the reference strain S288C, a comparison between the results from an ONT and a PacBio dataset with same read depth and similar read length distribution and accuracy is presented to assess the performance of the various pipelines in correcting the error types typical of the two technologies.

## Results

### Data Sequencing and Production

We have sequenced the genome of the four yeast strains, N44, SK1, CBS432 and S288C, with three different sequencing platforms: Illumina MiSeq, PacBio RS II, and MinION mk1. For each strain, we obtained about 80X depth of 2 × 150 bp MiSeq paired reads and between 120X and 250X depth from various PacBio runs. For the ONT runs, we obtained a total (‘Fail’ + ‘Pass’) 2D throughput (2D-All) between 12X and 61X depth depending on the strain, but only a throughput between 4X and 31X was achieved for the 2D-Pass data. Most Nanopore runs were performed using the R7.3 flowcells, available at the time of sequencing. For the reference strain S288C, the sequencing reads included two testing runs using the newly released R9 flowcell, in addition to the data of four runs from R7.3. The R9 flowcells generated data with higher accuracy than that of the R7.3 ones (91% against a R7.3 average of 88%). The R9 total 2D-All and 2D-Pass throughputs, 700 Mb and 60 Mb respectively, were too low for an independent study, so we merged the R7.3 and R9 reads to get a dataset of 61X depth 2D-All and a dataset of 31X depth 2D-Pass, upon which our assembly comparisons are based.

**Figure 1:**
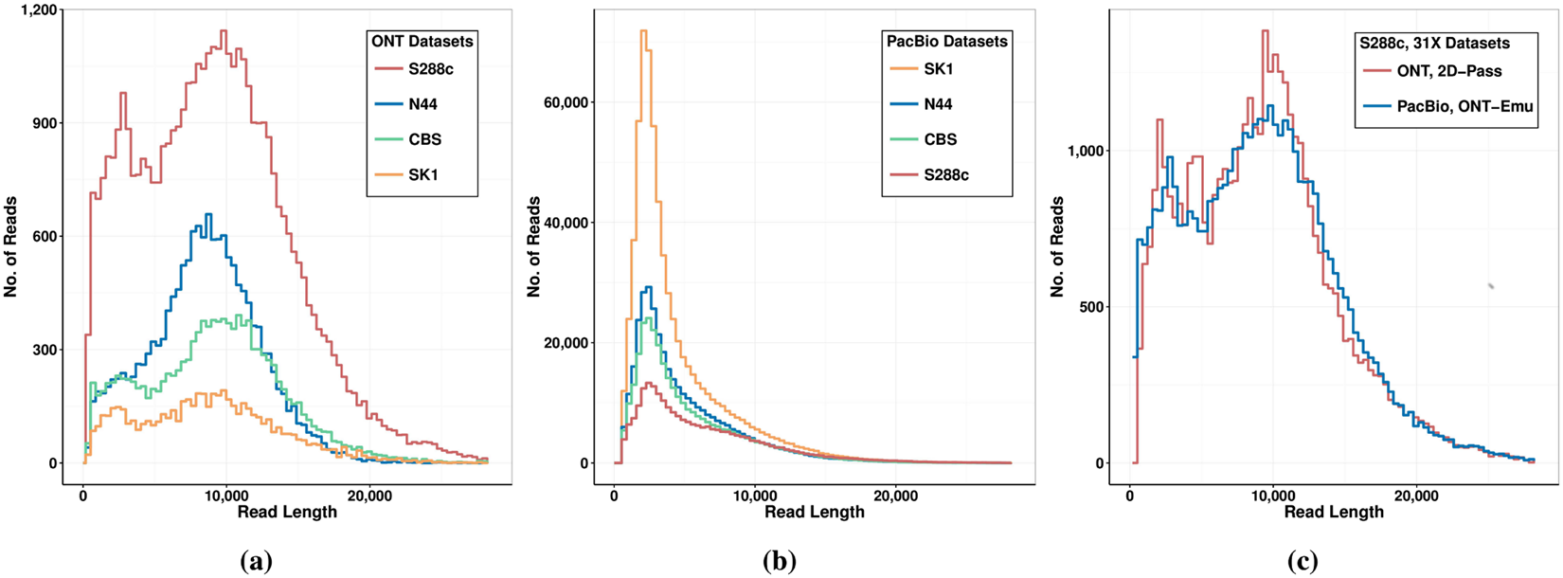
Read length distributions for the ONT and PacBio datasets. Read length distributions for the four yeast strains, S288C, N44, CBS432 and SK1 of the 2D-Pass ONT datasets in (**a**) and the PacBio datasets in (**b**). Comparison of read length distributions for the S288C strain of the 31X datasets ONT 2D-Pass and PacBio ONT-emulating 31X-subset in (**c**).

To compare the capabilities of the assembly pipelines we used the S288C strain data, so that assembly quality and accuracy could be easily and directly determined by mapping against its well known reference. Our S288C ONT and PacBio datasets have very different read depths: ONT only 31X (2D-Pass), while PacBio about 120X. By assembling the whole PacBio dataset we obtained contiguous assemblies with accuracy up to 99.95%, as shown in the Supplementary Note. For our pipeline comparison though, we decided to subset the PacBio dataset to the same depth of the ONT dataset, so that differences between platform results are driven by platform differences and not different sample sizes. If we selected the PacBio reads randomly until reaching the desired depth, the subset would follow a similar read length distribution of the original dataset, which has a smaller average read length than ONT. This would make it more difficult to interpret the assembly continuity as due to platform read intrinsic features like error rate and distribution, or different read lengths. Because of this, we decided to reduce the number of variables for assembly assessment by selecting a PacBio subsample of the S288C reads with the same depth (31X) but also similar read length distribution as the S288C 2D-Pass ONT reads: the PacBio ONT-Emu sample (Figure 1-(c)). More information on how we extracted the subset can be found in the Supplementary Note. To study the dependence of the assembly pipelines on read depth, we also selected two subsets with 10X and 20X depth for the S288C ONT and PacBio data, again with similar read length distributions. For the total PacBio S288C dataset, which consists of 120X read-depth, we performed a more detailed depth study shown in the Supplementary Note for subsets of 10X, 20X, 31X, 61X, 80X and the whole sample at 120X depth. Details about the sequenced datasets presented in this paper are summarized in Table 1 for ONT, and in Table 2 for PacBio samples which ‘emulate’ the ONT read length profiles (ONT-Emu). The read length distributions for the S288C, N44, CBS432 and SK1 strains of the 2D-Pass ONT datasets are shown in Fig. 1-(a) and those of the PacBio datasets in Fig. 1-(b).

**Table 1:** Statistic information for the 2D-Pass ONT datasets for the S288C, N44, CBS432 and SK1 strains.

Oxford Nanopore Datasets							
Strain	Dataset	Bases (Mb)	Reads	Average (b)	Longest (b)	N50 (b)	Identity
S288C	2D-Pass: 31X	383	42,325	9,040	56,477	11,693	93.3%
	2D-Pass: 20X	121	13,366	9,054	56,028	11,716	92.0%
	2D-Pass: 10X	242	26,721	9,057	56,477	11,659	92.8%
N44	2D-Pass: 11X	130	15,654	8,292	37,837	9,861	NA
CBS432	2D-Pass: 9X	110	12,211	8,952	46,481	11,201	NA
SK1	2D-Pass: 4X	51	5,938	8,589	36,791	10,971	NA

For the S288C strain, also shown are a 20X and a 10X subsets of randomly selected reads from the immediately larger 2D-Pass dataset.

**Table 2:** Statistic information for the PacBio datasets for the S288C, N44, CBS432 and SK1 strains.

Pacfic Biosciences Datasets							
Strain	Dataset	Bases (Mb)	Reads	Average (b)	Longest (b)	N50 (b)	Identity
S288C	120X	1,463	239,408	6,109	35,196	8,656	92.5%
	ONT-Emu: 31X	375	42,180	8,893	35,196	11,196	91.9%
	ONT-Emu: 20X	242	26,786	9,035	35,196	11,615	91.7%
	ONT-Emu: 10X	121	13,456	8,993	31,627	11,582	91.2%
N44	148X	1,794	371,025	4,834	33,906	6,800	NA
CBS432	135X	1,639	324,414	5,053	34,173	7,212	NA
SK1	248X	3,019	697,989	4,325	34,080	6,184	NA

For the S288C strain also the ONT-emulating subsets are shown: ‘ONT-Emu’ 31X, 20X and 10X subsets, selected to match the 31X, 20X and 10X ONT S288C datasets for depth and read length distribution.

### *De novo* Assembly and Scaffolding Pipelines

It has been shown already^12^ that contiguous and accurate yeast assemblies can be generated *de novo* solely using ONT data. Here, we focus on assessing the various existing pipelines and on comparing the results obtained from ONT and PacBio data with particular attention to the similarities and differences between the two platforms. The long reads provided by PacBio and ONT can be used to generate a *de novo* assembly either by themselves or in conjunction with Illumina (NGS) data. In this paper, we show examples of assemblies from long reads only, and from’hybrid’ pipelines that use the Illumina reads either to correct the long reads or to generate a short-read assembly that is later scaffolded with long reads.

We selected eight assembly pipelines for long reads. PBcR^13^ (with Self- or MiSeq-based error correction: PBcR-Self, PBcR-MiSeq), Canu^14^ and Falcon^15^ are based on an Overlap-Layout-Consensus (OLC) algorithm; ABruijn^16^ is based on a generalized De-Bruijn graph algorithm. All of them include a base-error correction step on the reads before assembling them. SMARTdenovo (available from https://github.com/ruanjue/smartdenovo) is also based on a OLC algorithm, but does not include a base-error correction step. Miniasm^17^ chooses the best path from a string graph created from the overlap of uncorrected reads: it is the only pipeline here that includes neither a base-error correction nor a consensus step. Racon^18^ aligns the raw long reads to a Miniasm assembly and generates a consensus, significantly increasing the initial accuracy. We selected and assessed also three scaffolding pipelines: npScarf^19^, HybridSPAdes^20^ and SMIS (available from https://github.com/fg6/smis.git), to scaffold an NGS-based assembly from SPAdes^21^. Details about these pipelines and the parameters used for running them can be found in the Supplementary Note.

### Assemblies from different pipelines and platforms

The *de novo* assemblies of the whole PacBio datasets for S288C and the other strains are shown and discussed in the Supplementary Note, while here we compare the performances of long-read assemblers when run on a 31X depth ONT or PacBio datasets with similar read length distributions. For this purpose we selected a subsample from the PacBio S288C dataset with the same depth as the ONT 2D-Pass sample (31X) and with similar read length distribution, as described above and shown in Fig. 1-(c). The statistic information for the two S288C samples can be compared if looking at the sample S288C ‘2D-Pass: 31X’ in Table 1 for the ONT case, and at the S288C ‘ONT-Emu 31X’ in Table 2 for the PacBio case emulating the ONT sample. As shown in these tables, the two samples also have similar average error rates (92% average identity for PacBio and 93% for ONT datasets). Using these two similar samples, except for normal fluctuations, differences in assembler’s performances can be attributed to the peculiar features of the reads from the two platforms, for instance to error types and distributions.

The assembly information for the ONT dataset ‘2D-Pass 31X’ and for the PacBio ‘ONT-Emu 31X’ can be found in Tables 3 and 4, respectively. With 31X depth the pipelines generated assemblies with similar features when running on PacBio or ONT data.

**Table 3:** Statistic information about the *de novo* assemblies for the S288C ONT datasets for the hybrid pipeline PBcR-MiSeq, the pipeline Miniasm, with no base error correction nor consensus step, and for the non-hybrid pipelines: Racon (on a Miniasm draft assembly), Falcon, SMARTdenovo, ABruijn, PBcR-Self and Canu for, from top to bottom: all the 2D-Pass reads (2D-Pass 31X), the 2D-Pass 20X subset and the 2D-Pass 10X subsets.

Oxford Nanopore S288C Datasets												
Dataset	Assembler	Bases (Mb)	Contigs	N50 (kb)	Reference Coverage	SNPs, Indels (#per kb)	Identity	MisAss	Na50 (kb)	Genes (6,615)	CPU Time (h)	Memory (GB)
2D-Pass 31X	PBcR-MiSeq	11.9	76	305	99.08%	**0.1, 0.2**	**99.94%**	**18**	**273**	**6,514**	**147**	**17**
	Miniasm	11.8	27	739	94.85%	34, 67	89.42%	26	362	3,353	**0.1**	**5**
	Racon	12.0	27	752	98.80%	0.4, 11	**98.76%**	24	534	6,533	8	5
	Falcon	11.9	43	717	99.09%	0.5, 21	97.79%	27	**546**	6,526	**19**	**71**
	SMARTdenovo	**12.1**	28	625	99.54%	0.3, 14	98.50%	25	531	6,556	2	**5**
	ABruijn	12.4	**26**	**769**	98.89%	**0.1**, 15	98.49%	31	536	6,533	44	8
	PBcR-Self	12.9	64	616	99.21%	0.2, 17	98.24%	92	525	6,552	695	23
	Canu	**12.1**	29	698	**99.62%**	**0.1**, 17	98.30%	34	530	**6,566**	80	14
	+Nanopolish	12.3	29	709	99.63%	0.1, 4	99.57%	35	538	6,584	1,835	12
2D-Pass 20X	PBcR-MiSeq	11.8	66	269	99.09%	**0.1**, **0.2**	**99.94%**	**8**	262	6,522	95	13
	Miniasm	11.6	39	418	94.66%	34, 67	89.36%	24	286	3,271	**0.1**	**3**
	Racon	11.8	39	423	98.11%	0.7, 13	**98.56%**	26	393	6,478	5	**2**
	Falcon	10.7	84	210	90.64%	0.6, 21	97.56%	17	194	5,946	10	44
	SMARTdenovo	11.9	**29**	**656**	98.99%	0.8, 16	98.23%	24	455	6,528	1	4
	ABruijn	**12.0**	**29**	468	98.55%	0.3, 16	98.28%	12	436	6,495	29	7
	PBcR-Self	12.9	72	545	**99.32%**	0.3, 18	98.08%	74	452	**6,550**	342	20
	Canu	11.9	31	544	98.99%	0.2, 18	98.10%	25	441	6,525	41	10
2D-Pass 10X	PBcR-MiSeq	11.3	123	**161**	**95.75%**	**0.1, 0.2**	**99.94%**	13	**146**	**6,310**	33	7
	Miniasm	7.9	158	58	67.90%	24, 46	89.26%	12	43	2,256	0.02	0.002
	Racon	8.1	158	60	70.33%	2, 13	97.72%	15	58	4,520	3	1
	Falcon	1.4	113	17	15.90%	0.1, 3	97.43%	6	16	901	3	24
	SMARTdenovo	10.4	**114**	115	88.71%	5, 24	96.58%	**12**	104	5,610	**1**	**1**
	ABruijn	8.5	86	111	72.68%	1, 16	97.47%	21	97	4,711	16	8
	PBcR-Self	**11.5**	167	106	91.33%	1, 22	**97.40%**	64	102	5,957	71	3
	Canu	10.7	115	134	91.52%	1, 23	97.32%	18	112	5,955	13	6

For the 2’D-Pass 31X’ dataset also the results from Nanopolish on the Canu assembly is shown. In each column the best value is highlighted in bold. For the identity column the best value is always for the hybrid assembly PBcR-MiSeq, but we also highlighted (bold and underlined) the best value for the non-hybrid pipelines, ignoring Nanopolish as it is the only polishing tool. For the 10X datasets, we ignored assemblies with less than 80% reference coverage when choosing the best values.

**Table 4:** As Table 3 but for PacBio-based assemblies from the ONT-Emu PacBio subsets at, from top to bottom, 31X, 20X and 10X depth.

Pacific Biosciences S288C Datasets												
Dataset	Assembler	Bases (Mb)	Contigs	N50 (kb)	Reference Coverage	SNPs, Indels (# per kb)	Identity	MisAss	Na50 (kb)	Genes (6,615)	CPU Time (h)	Memory (GB)
ONT-Emu 31X	PBcR-MiSeq	11.9	76	270	98.68%	**0.1, 0.1**	**99.97%**	**9**	270	6,526	132	17
	Miniasm	12.5	35	563	96.10%	19, 88	89.37%	53	106	3,226	**0.1**	**5**
	Racon	**12.1**	34	544	99.09%	0.3, 4	99.50%	22	429	6,540	19	5
	Falcon	12.0	35	549	98.18%	0.3, 2	99.78%	28	436	6,508	13	64
	SMARTdenovo	12.3	**20**	**929**	**99.97%**	0.2, 3	99.66%	27	549	6,596	2	**4**
	ABruijn	12.3	26	666	99.30%	**0.1**, 1	99.87%	43	469	6,565	19	7
	PBcR-Self	12.4	39	751	99.50%	**0.1**, 1	99.92%	43	548	6,590	63	24
	Canu	12.3	28	607	99.92%	**0.1**, 1	**99.93%**	29	534	**6,601**	15	10
ONT-Emu 20X	PBcR-MiSeq	11.7	64	304	98.73%	**0.1, 0.1**	**99.97%**	**7**	264	6,501	86	13
	Miniasm	12.0	86	202	93.08%	18, 84	89.57%	53	69	3,255	**0.04**	**3**
	Racon	11.6	86	194	95.35%	1, 7	99.18%	25	189	6,241	10	**2**
	Falcon	9.9	152	115	82.22%	0.3, 2	99.65%	29	112	5,341	5.6	41
	SMARTdenovo	**12.2**	**38**	**545**	**99.80%**	**0.5, 8**	99.09%	24	434	6,534	1	3
	ABruijn	11.7	58	272	96.06%	**0.1**, 2	99.72%	36	258	6,325	23	9
	PBcR-Self	12.3	44	502	99.03%	0.2, 2	99.78%	35	428	6,560	30	20
	Canu	**12.2**	42	454	99.47%	0.2, 2	**99.79%**	28	432	**6,565**	8	7
ONT-Emu 10X	PBcR-MiSeq	**10.9**	**144**	**117**	**92.26%**	**0.1, 0.1**	**99.97%**	**9**	**111**	**6,058**	**46**	**7**
	Miniasm	4.0	120	35	33.92%	6, 27	89.61%	4	19	1,035	0.02	0.1
	Racon	3.8	120	34	35.48%	1, 5	98.28%	8	33	2,095	5	1
	Falcon	0.6	59	14	8.99%	0.1, 0.2	99.41%	10	13	421	1	23
	SMARTdenovo	8.5	157	61	71.29%	3, 22	96.43%	8	55	4,271	1	1
	ABruijn	4.7	67	71	41.45%	0.4, 4	98.87%	10	67	2,631	13	7
	PBcR-Self	9.7	232	57	78.99%	1, 7	98.96%	35	55	5,111	12	18
	Canu	8.9	178	62	75.35%	0.4, 6	99.14%	19	59	4,811	3	4

The fastest pipeline is Miniasm, which does not include a base error correction nor a consensus step. For both PacBio and ONT datasets it only took 4–5 minutes to run but only achieved 89% accuracy. Because of the high number of indels in the ONT and PacBio data, Miniasm reconstructed only about 95–96% of the genome, significantly less than the other pipelines. Globally, the assembly structure from the Miniasm pipeline is correct, indicating that the missing regions are due to local base errors. Part of these base errors are recovered by the other pipelines either by building a consensus from the raw reads (Racon, SMARTdenovo) or during their base-error correction steps (PBcR-MiSeq, PBcR-Self, Canu, Falcon and ABruijn). The assemblies with the highest accuracy, 99.94–99.97% are the ones from the only hybrid assembler, PBcR-MiSeq, that uses MiSeq reads to correct the ONT or PacBio reads. But PBcR-MiSeq also provided the most fragmented assemblies, with Na50s only 270 kb long, where Na50s are the N50s after breaking the contigs at the misassembly points found by Quast^22^. None of the other assemblers uses Illumina reads, and we refer to them as the non-hybrid pipelines. The non-hybrid pipelines reconstructed 98–99% of the genome, with Na50s in the 400–500 kb range long, and accuracy up to 98.76% for ONT reads and up to 99.93% for the PacBio reads. The highest accuracy between the non-hybrid assemblies for the ONT data is from Racon, with 98.76%, immediately followed by SMARTdenovo and ABruijn with about 98.50%. For the PacBio datasets the Celera-based assemblers (Canu, PBcR-Self) provided the highest accuracy, 99.92–99.93%, followed by ABruijn at 99.87% and Falcon at 99.78%.

To assess the completeness of the newly generated assemblies, we checked for the reconstruction of a list of known S288C genes. From the list of ORF coding sequences reported in the Saccharomyces Genome Database (http://www.yeastgenome.org), we selected 6,615 coding sequences that mapped to the S288C reference for at least 90% of their length with at least 90% mapping identity. We used BWA^23^ to align each gene against the new assembly under study, and declared a gene ‘reconstructed’ if at least 90% of it was assembled with at least 90% accuracy. Tables 3 and 4 for ONT and PacBio data respectively, show a correlation between the assembly continuity and the number of mapped genes, i.e. an assembly from a particular pipeline with longer contigs is likely to have reconstructed more genes. For example, the SMARTdenovo assemblies consist of 20 contigs when using 31X PacBio reads and 38 contigs when using 20X PacBio data, and the numbers of reconstructed genes are 6,596 and 6,534 respectively. For the ONT data at 31X, the contig number is 28, and it is associated with 6,556 genes; at read coverage of 20X, the contig number is increased to 29, while mapped genes dropped to 6,528.

Even though there is no single pipeline that outperforms at every statistic gathered, SMARTdenovo and Canu assemblies have the longest reference coverage, best or near-best average identity, highest number of genes found and long Na50s for both PacBio and ONT data. Apart from Miniasm, SMARTdenovo is also the pipeline using the least resources, with CPU running time ≤2 h and ≤5 GB of memory. Racon, Falcon and ABruijn are the next fastest pipelines. PBcR is the pipeline typically requiring the longest running times especially when using ONT data.

### Missing homomers

**Figure 2:**
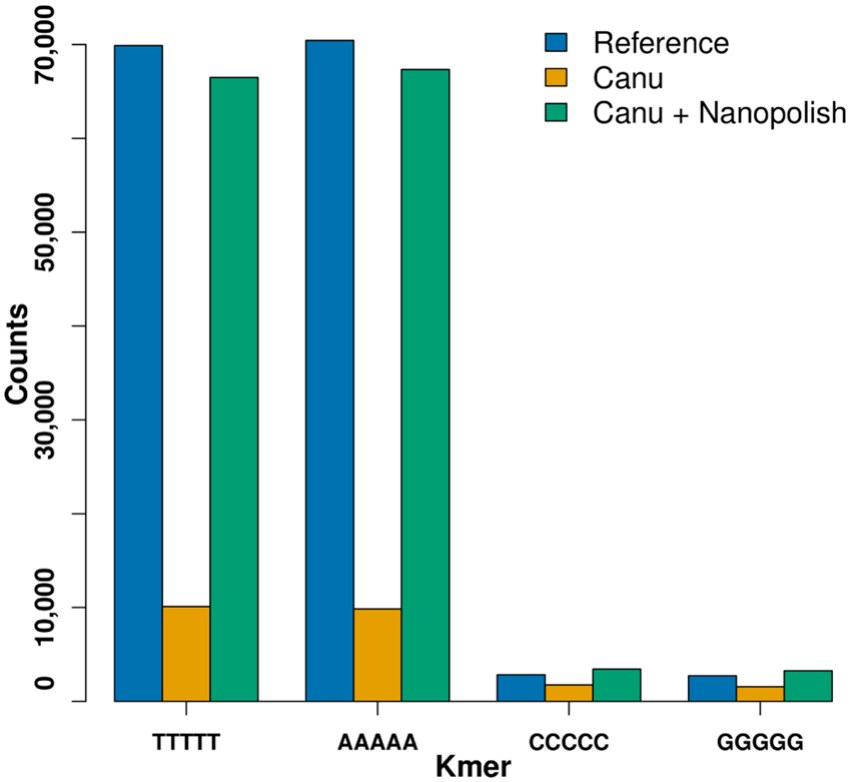
Homomer counts. Counts for the 5 bases homomers in the Reference (blue), in the Canu assembly (orange), and in the Canu assembly after polishing with Nanopolish (green).

Ignoring Miniasm which has no error correction nor a consensus step, and the hybrid pipeline PBcR-MiSeq, which corrects the reads using Illumina data, it is clear that the PacBio assemblies have higher accuracies, from 99.50% to 99.93%, while the ONT assemblies reach a maximum accuracy of 98.76%. Because the average read accuracy, the depth and the read length distribution of the ONT and PacBio datasets used here are very similar, the final higher accuracies for the PacBio assemblies are likely due to platform-intrinsic features of the data. When no base-error correction nor consensus step is performed, like in the Miniasm pipeline, the resulting assemblies are about 89% accurate for both the ONT and PacBio datasets, and present lots of mismatches and indels, with prevalence of indels. Adding a correction or a consensus step like the other non-hybrid pipelines significantly reduces the number of mismatches and indel errors for both ONT and PacBio data. While the number of mismatches is reduced to a similar level for ONT and PacBio, the number of indels in ONT assemblies remains very high. The ONT assembly with fewer indels (Racon, indels = 11 per kb) has about 11 times more indels than the best PacBio case (Canu, indels = 1 per kb), and about 3 times more indels than the worst PacBio case (Racon, indels = 4 per kb). The high accuracy reached with the PacBio data shows that most PacBio errors can be corrected by generating a consensus between the reads that cover the same genomic region, and is a clear indication that PacBio read errors are mostly randomly distributed. For the ONT data the situation is more challenging: while it is true that some of the errors have a random distribution and are corrected by the various pipelines, a good portion of them, mainly indels, seem to escape the correction attempts, pointing to a possible systematic source of errors. Indeed most of the remaining errors are due to missing homomers in the ONT reads. For instance, the assembly from Canu contains far fewer 5-homomers (“AAAAA”, “TTTTT”, “CCCCC” and “GGGGG”) than the reference, as shown in Fig. 2, where the blue bars represent the Reference’s counts and the orange bars Canu’s counts. The figure also shows that if the Canu assembly is polished with Nanopolish^24^ (green bars) many of the missing homomers are recovered, and the final assembly accuracy reaches 99.57%, as shown in Table 3 for the ‘2D-Pass 31X’ sample. This table also shows the downside of Nanopolish: it needs 1,835 CPU hours to polish the Canu assembly, which makes this pipeline impractical for larger genomes.

### Mitochondrial genome reconstruction

While all of the 16 yeast nuclear chromosomes are well reconstructed by every tested pipeline with respect to continuity, accuracy and completeness, many pipelines failed to reconstruct the mitochondrial genome (Supplementary Tables S5 and S6). This chromosome is the smallest one in the reference assembly, only containing 85,779 bases, but appears to be very challenging to assemble, especially with ONT datasets. Using ONT data, only PBcR-MiSeq was able to reconstruct it almost completely (96%), and only Falcon and Canu were able to reconstruct at least half of it (67%, 64%, respectively). Using PacBio data there are three full reconstructions from Racon, SMARTdenovo and Canu, while Miniasm manages to reconstruct 77% of it. There are a number of possible reasons for the mitochondrial genome to be more challenging to reconstruct than the other chromosomes. The GC content in the mitochondrial genome is only 17%, much lower than the average value of 38% for the nuclear chromosomes. There are also a lot of highly repetitive small AT *k*-mers, which contribute to the difficulty of assembly.

We found that while for the ONT dataset the mitochondrial read depth was consistent with that of the other chromosomes, the PacBio mitochondrial depth was about 6 times higher which could help explain why the mitochondrial genome reconstruction is more complete with the PacBio data. To exclude the possibility that this is due to a bias in our subsetting, we looked at the whole PacBio dataset and found that, similarly to the subset, the mitochondrial genome depth is about 7 times higher than the nuclear chromosomes. This is likely due to the high copy number of mitochondria in a cell. Possibly, the PacBio library preparation preserves this higher copy number, while the ONT library preparation does not. For instance, it is possible that DNA molecules have been less vigorously sheared in the ONT case, so that many of the very short mitochondrial genome remained circular preventing the ligation of the adapters needed for sequencing.

**Figure 3:**
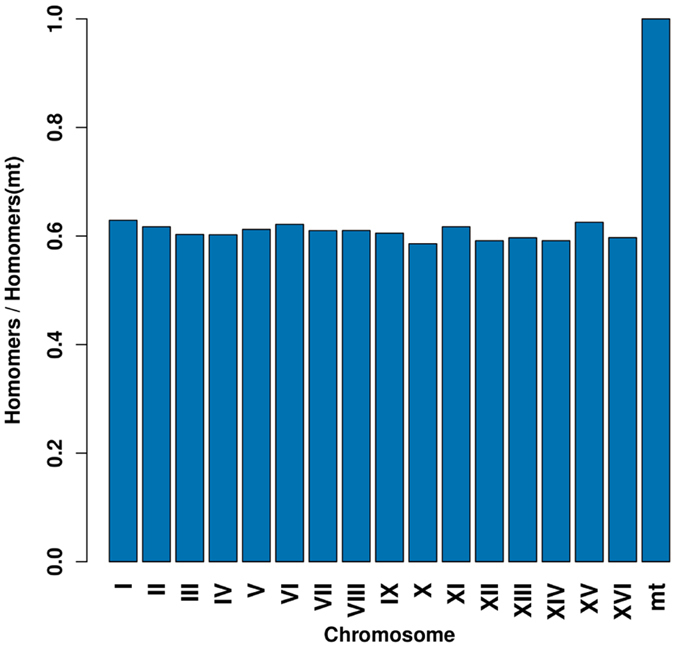
Chromosome homomer rate with respect to that of the mitochondrial genome. Ratios of 5–homomer counts normalized by the chromosome length between each chromosome and the mitochondrial genome (mt).

In addition to a lower depth with respect to PacBio, the mitochondrial genome assembly from ONT data is also hindered by an higher than average homomer content: using the S288C reference, we estimated that the mitochondrial genome has at least 30% more homomers than the other chromosomes, for *k*-mer lengths 3, 4, 5, 6 and 7. Figure 3 shows the 5-homomer counts (#5A + #5T + #5G + #5C) ratios between each chromosome and the mitochondrial genome, each count normalized by the chromosome length in bases.

### *De novo* Assembly by Varying the Read Depth

In order to assess the scalability of the assembly pipelines and to determine how the performances of the assemblers vary with read depth, we selected and analyzed subsets of the ONT and the PacBio full datasets. A dedicated study focusing only on the higher depth PacBio dataset is discussed in the Supplementary Note. Here, we compared the assemblies at 31X mentioned in the previous section, with 20X and 10X subsets from the ONT 2D-Pass and similar subsets from PacBio reads emulating the read length distribution of the ONT subsets, as we did for the 31X case. These datasets are presented in Tables 1 and 2 for ONT and PacBio respectively, where the PacBio subsets are labeled as ONT-Emu. The results of the pipelines are summarized for the 31X, 20X and 10X in Table 3 for ONT and Table 4 for PacBio data. At very low read depth, 10X, the hybrid pipeline, PBcR-MiSeq, was the best performing pipeline for both ONT and PacBio data providing assemblies which cover more than 92% of the genome with accuracies larger than 99.9%, and the longest Na50s, in the 100s of kb. At 20X depth the Na50 is about doubled, and 98–99% of the genes are reconstructed.

For the non-hybrid pipelines, the Celera-based ones, Canu and PBcR-Self, were the best performers at low read depths. At 10X, Canu and PBcR-Self performed better on ONT than PacBio data. These assemblies cover about 90% (75–79%) of the reference with an accuracy of 97% (99%) and a Na50 of 100 (60) kb with ONT (PacBio) data. All the other assemblies cover significantly less proportions of the reference genome, and in particular Falcon seems to have the most difficulties at such low read depth. At 20X, all the Celera-based pipelines still performed very well, with Canu and PBcR-Self providing Na50s in the range of 400s kb, as well as SMARTdenovo. The assembly from Racon distinguishes itself on ONT data for the highest accuracy, immediately followed by ABruijn and SMARTdenovo, while for PacBio data Canu, PBcR-Self and ABruijn assemblies have the highest accuracies. Already at 20X, Canu, PBcR-Self, PBcR-MiSeq and SMARTdenovo reconstructed about 99% of the genes. At 31X depth the non-hybrid pipelines increased the Na50s up to 500s kb and slightly improved their final accuracies.

### Genome scaffolding using long reads

We explored the scaffolding performance of three pipelines that use long ONT/PacBio reads to bridge and merge contigs from a NGS assembly. The NGS assembly used here has been generated from a dataset of 80X of 2 × 150 bp MiSeq paired-reads by SPAdes, as one of the scaffolding pipelines is embedded with it (HybridSPAdes). As expected, the assembly from SPAdes is fragmented but has very high accuracy: it has 206 contigs and its N50 is only 125 kb, as shown at the top of Table 5. The scaffolding pipelines were able to bridge a number of contigs significantly increasing the assemblies’ N50s while maintaining a high accuracy for both the PacBio and ONT samples. The npScarf pipeline achieved N50s much longer than HybridSPAdes or SMIS, 771 kb for ONT and 715 kb for PacBio, but the Quast analysis of these assemblies revealed that they are affected by a number of misassemblies, and their final Na50 is 514 kb for the ONT data and 413 kb for PacBio. Even after correcting for the misassemblies npScarf provided the more contiguous assemblies, but the HybridSPAdes and SMIS ones were able to maintain a higher accuracy than npScarf, 99.97–99.98% against 99.91%. The assemblies from HybridSPAdes have a higher coverage over the reference and reconstruct more genes than SMIS or npScarf. The npScarf tool has the advantage of requiring the least resources as it is faster than the other two pipelines and it uses less memory.

**Table 5:** MiSeq-only assembly from SPAdes in top row.

S288C Datasets: Scaffolding Pipelines												
Dataset	Assembler	Bases (Mb)	Contigs	N50 (kb)	Reference Coverage	SNPs, Indels (# per kb)	Identity	MisAss	Na50 (kb)	Genes (6,615)	CPU Time (h)	Memory (GB)
MiSeq	SPAdes	11.6	206	125	98.3%	0.04, 0.03	99.98%	5	125	6,399	5	12
ONT 2D-Pass 31X	npScarf	**11.9**	**21**	**771**	99.8%	0.4, 0.3	99.91%	69	**514**	6,559	**3**	**4**
	HybridSPAdes	11.8	64	444	**99.97%**	0.1, **0.04**	99.97%	**7**	416	**6,582**	18	12
	SMIS	11.8	85	549	98.4%	**0.04, 0.04**	**99.98%**	13	493	6,411	13	**4**
PacBio ONT-Emu 31X	npScarf	**11.7**	**22**	**715**	98.5%	0.3, 0.4	99.91%	67	**413**	6,458	**2**	**3**
	HybridSPAdes	**11.7**	68	364	**99.9%**	0.1, **0.04**	**99.97%**	**5**	317	**6,583**	27	12
	SMIS	**11.7**	89	546	98.8%	**0.04, 0.04**	**99.97%**	40	309	6,399	9	6

MiSeq-only assembly from SPAdes scaffolded by the npScarf, HybridSPAdes and SMIS pipelines using the ‘2D-Pass 31X’ ONT sample (Middle) and the ‘ONT-Emu 31X’ PacBio subset (Bottom).

We ran the same scaffolding pipelines on the three other yeast strains N44, CBS432 and SK1 with 2D-Pass ONT data at the lower depths of 11X, 9X and 4X, respectively. The scaffolding results are shown in Table 6. As a reference is not available for these strains, we could not evaluate the accuracy and misassemblies of the scaffolds, but, as for the S288C case, we can expect high accuracy, >99.95%, as these assemblies are based on Illumina data. The longer N50s from npScarf are likely significantly affected by misassemblies, as observed in the S288C case, while the N50s estimated by HybridSPAdes and SMIS are expected to be more accurate. A comprehensive structure variation analysis of these strains can be found in ref. 25.

**Table 6:** Statistic information on the *de novo* assemblies from the MiSeq-only SPAdes pipeline and the same SPAdes assembly scaffolded with npScarf, HybridSPAdes and SMIS for the N44 (top panel), CBS432 (middle panel), and SK1 (bottom panel) strains.

Oxford Nanopore Datasets							
Dataset	Assembler	Bases (Mb)	Contigs	N50 (kb)	Genes (6,615)	CPU Time (h)	Memory (GB)
N44 2D-Pass: 11X	SPAdes	11.6	187	117	5,475	7	13
	npScarf	11.7	19	898	5,538	1	2
	HybridSPAdes	11.7	61	324	5,547	8	13
	SMIS	11.7	58	511	5,474	2	5
CBS432 2D-Pass: 9X	SPAdes	11.6	181	150	5,498	5	12
	npScarf	11.4	19	928	5,443	1	1
	HybridSPAdes	11.7	49	515	5,611	6	12
	SMIS	11.7	64	658	5,499	1	5
SK1 2D-Pass: 4X	SPAdes	11.6	240	118	6,341	6	12
	npScarf	11.7	43	507	6,435	0.3	1
	HybridSPAdes	11.7	111	227	6,444	5	12
	SMIS	11.7	142	358	6,341	1	3

## Discussion

We have shown that the yeast genome can be *de novo* assembled with Na50s up to 550 kb with 31X read depth from PacBio or ONT platforms, reaching an accuracy of up to 99% for PacBio and 98% for ONT, when not using Illumina data. More fragmented assemblies but with an higher accuracy (up to 99.98%) can be achieved when using long reads in conjunction with Illumina reads in hybrid or scaffolding assemblies. Miniasm was the fastest pipeline, requiring only few minutes to run, but because of the lack of a correction or consensus step it generated assemblies with very poor accuracy (~89%). After Miniasm, SMARTdenovo is the pipeline requiring the least resources and in particular the least time for running, <2 h, but providing accuracies close to the highest one achieved without help from Illumina (98.5% for ONT and 99.7% for PacBio). The non-hybrid Celera-based assemblers, PBcR-Self and Canu, generated continuous and accurate assemblies, and appeared to be the most performing pipelines as the read coverage decreased.

In our experience, PacBio and ONT platforms provided reads with similar error rates and lengths in the thousands of bases. The average PacBio lengths were generally smaller than the ONT ones, but the ONT throughput per run was significantly lower than the one from PacBio’s runs. This was mainly because we used early MinION flowcells and chemistries: the throughput improved significantly both in size and stability with more recent MinION kits and software. In addition, a fairer throughput comparison should be done between two benchtop devices, *e.g*. the PacBio RSII and the Oxford Nanopore GridION, or PromethION, both not available at the time of this study. Both platforms provided error-prone reads, but missing homomers in the ONT data represent a major difference with respect to PacBio. Because of this difference, while PacBio reads could be corrected with enough depth to reach assembly accuracies >99.9%, for ONT data increasing the read depth only helped to reach an accuracy up to 98–99%. To improve further a very time consuming polishing step is needed, at least until the missing homomers issue is reduced or solved, possibly already with a new Neural Network-based basecaller, Scrappie, presently in development at Oxford Nanopore.

## Methods

### Library Preparation

#### Library preparations for Oxford Nanopore sequencing

Two ug of genomic DNA was sheared to approximately 18,000 bp by centrifugation at 4000 rpm in a gTUBE. Sequencing libraries were prepared according to the SQK-MAP006 or SQK-NSK007 Sequencing Kit protocol, including the NEBNext FFPE DNA repair step.

#### MinION^TM^ flow cell preparation and sample loading

The sequencing mix was prepared with 6 uL of the DNA library, water, the Fuel Mix and the running buffer according to the SQK-MAP006 or the SQK-MAP007 protocols. The sequencing mix was added to the R7 or R9 flowcell for a 24–48 hour run. Typically the flowcells were reloaded after 24 hours as data yield had plateaued.

#### Pacific Biosciences sequencing library preparation

PacBio sequencing libraries were prepared as follows. Five ug of genomic DNA was sheared to approximately 15,000 bp by centrifugation at 5200 rpm in a gTUBE. DNA was repaired with damage repair reagent and end-repaired using end repair mix before ligation to PacBio blunt end adapter. Unligated material was digested with Exo III and Exo VII then library fragments purified via two consecutive Ampure clean-ups and size selection on Blue Pippin (SageScience, Beverley, MA, USA) with a 0.75% agarose cassette to purify fragments from 12–25 kb.

#### Illumina PCR-free library preparation and sequencing

DNA (1 ug) was sonicated to a 400 to 600 bp size range using a Covaris LE220 acoustic shearing device (Covaris, Woburn, MA, USA). Fragments were end-repaired using the NEBNext EndRepair Module (New England Biolabs, Ipswich, MA, USA) and A-tailed with the NEBNext dA-Tailing Module. Illumina adapters were added using the NEBNext Quick Ligation Module. Ligation products were purified with AMPure XP beads (Beckman Coulter Genomics, Danvers, MA, USA). Libraries were quantified by qPCR using the KAPA Library Quantification Kit for Illumina Libraries (Kapa Biosystems, Wilmington, MA, USA) and library profiles were assessed using a DNA High Sensitivity LabChip kit on an AgilentBioanalyzer (Agilent Technologies, Santa Clara, CA, USA). Libraries were sequenced on an Illumina MiSeq instrument (San Diego, CA, USA) using paired 150 base read chemistry.

### Assembly Assessment and Other Tools

The ONT fastq sequencing reads were extracted from the fast5 files using Poretools^26^. For the assembly accuracy and other summary statistics we used dnadiff from MUMmer^27^, while the number of misassemblies and the Na50s, i.e. the N50s after breaking the contigs at the misassembly points, were calculated using Quast^22^. We ran Quast with default parameters except for the Miniasm assemblies. For Miniasm we required the additional parameter: –min-identity 85, to enable Quast to align the low accuracy assemblies to the reference. Because of such low accuracies, Quast seemed to overestimate the presence of misassemblies for Miniasm, and reported Na50s smaller than the expected ones if comparing the Na50s with those of the higher accuracy Racon assemblies, based on Miniasm. We used the R Biostrings package (available from https://bioconductor.org/packages/release/bioc/html/Biostrings.html) to count the number of homomers in some of the assemblies, using the function oligonucleotideFrequency. Statistics and assessment for all the assemblies have been estimated after eliminating contigs shorter than 1 kb.

## Electronic supplementary material

Supplementary Note
