# Supplementary material for: De novo yeast genome assemblies from MinION, PacBio and MiSeq platforms
Source: Sci Rep. 2017 Jun 21;7:3935. doi: 10.1038/s41598-017-03996-z (PMC5479803; doi:10.1038/s41598-017-03996-z)
Supplement: Supplementary file 1 — Supplementary Note [file 41598_2017_3996_MOESM1_ESM.pdf]

De novo yeast genome assemblies from MinION, PacBio and MiSeq platforms
Supplementary Materials

Francesca Giordano[1,*], Louise Aigrain[1], Michael A. Quail[1], Paul Coupland[2], James K. Bonfield[1], Robert M. Davies[1], German Tischler[3], David K. Jackson[1], Thomas M. Keane[1], Jing Li[4], Jia-Xing Yue[4], Gianni Liti[4], Richard Durbin[1], and Zemin Ning[1]

[1]The Wellcome Trust Sanger Institute, Wellcome Trust Genome Campus, Hinxton, Cambridge, UK
[2]Cancer Research UK Cambridge Institute, Li Ka Shing Centre, University of Cambridge, Cambridge CB2 0RE, UK
[3]Max Planck Institute of Molecular Cell Biology and Genetics , Pfotenhauerstraße 108, 01037 Dresden, Germany
[4]Université Côte d'Azur, CNRS, INSERM, IRCAN, Nice, France
[*]Correspondence should be addressed to F.G. (email: francesca.giordano@sanger.ac.uk)

De novo Assembly and Scaffolding Pipelines

The *de novo* assembly pipelines we assessed and the parameters we used are listed in this section. Scripts to download the data and run these pipelines are available from GitHub: https://github.com/fg6/YeastStrainsStudy.git.

- **Miniasm.** Miniasm is a fast assembler developed for long and error-prone reads. An assembly graph is generated from overlapping reads found by Minimap, a MinHash-sketch-based aligner[1]. Small bubbles are collapsed and unitigs are built from the graph, without any error correction step nor a consensus generation from the aligned reads. We ran MiniMap version r122 and Miniasm version r104 using default settings and parameters.

- **Racon.** Racon aligns the long reads to a low accuracy draft assembly and improves the quality of the assembly by generating a consensus from the aligned reads. We tested Racon (github commit 28980bec3e98189853ed919764d5a8a9e6291264) on a Miniasm assembly generated as in the previous point. The time and memory consumption reported for Racon include the resources used to generate the initial Miniasm assembly. The raw reads (reads.fasta) were aligned to the Miniasm assembly (contigs.fasta) using GraphMap[2]:

  ```
  $ graphmap align -a anchor --rebuild-index -B 0 -r contigs.fasta \
         -d reads.fasta -o output.sam --extcigar -t 8
  ```

  and the consensus was generated with Racon in the following way:

  ```
  $ racon -M 5 -X -4 -G -8 -E -6 --bq 10 -t 8 contigs.fasta \
         output.sam consensus.fasta
  ```

  We ran a second iteration of consensus generation by realigning the raw reads against the consensus from the first iteration of Racon and by generating a new consensus in the same way as in the first iteration.

- **Falcon.** Falcon was originally developed to correct and assemble PacBio data, but it can also be used with ONT data. Before assembling the long reads, it corrects them by generating a consensus from an all-versus-all alignment obtained from a modified version of DALIGNER (https://github.com/cschin/DALIGNER). We ran Falcon release v0.3.0 with the parameters:

  ```
  avoid_text_file_busy=true
  length_cutoff = 1000
  length_cutoff_pr = 1000
  pa_concurrent_jobs = 30
  ovlp_concurrent_jobs = 30
  pa_HPCdaligner_option =  -v -dal4 -t16 -e.70 -l100 -s100
  ovlp_HPCdaligner_option = -v -dal4 -t30 -h60 -e.92 -l100 -s100
  pa_DBsplit_option = -x500 -s50
  ```

```
ovlp_DBsplit_option = -x500 -s50
falcon_sense_option= --output_multi --min_idt 0.50 --max_n_read 200 --n_core 8
overlap_filtering_setting=--max_diff 100 --max_cov 100 --min_cov 5 --bestn 20 --n_core 8
```

- **PBcR.** PBcR is a Celera based assembler[3]. Before assembly, PBcR has a read error correction step. The error correction can be performed either with or without Illumina reads. When using Illumina reads, the short reads are aligned by the Celera built-in aligner against the long reads that are corrected accordingly. Without Illumina reads, long reads are aligned all-against-all and a consensus is generated (self-correction). In this last case, we chose to use the MHAP aligner[4], which is faster and performs better than the other possible choice, BLASR (https://github.com/PacificBiosciences/blasr). We showed both options for correction, referred to as PBcR-MiSeQ and PBcR-Self, respectively. We used PBcR from the Celera Assembler package version 8.3rc2. The spec file pbcr.spec included the following parameters for all datasets and for the MiSeQ and Self cases:

```
merSize=16
frgCorrThreads = 10
useGrid=0
scriptOnGrid=0
ovlMemory=50
ovlStoreMemory=100000
threads=20
ovlConcurrency=1
cnsConcurrency=8
merylThreads=32
merylMemory=32000
frgCorrBatchSize = 100000
ovlCorrBatchSize = 100000
```

For the Self case the spec file included the additional parameters:

```
mhap=-k 16 --num-hashes 512 --num-min-matches 3 --threshold 0.04 --weighted
ovlRefBlockSize=20000
```

and PBcR was run with the command:

```
$ PBcR -length 500 -partitions 200 -l S288c -s pbcr.spec  -fastq reads.fastq  \
  genomeSize=12160000
```

For the MiSeq case, before running PBcR we generated a Celera Assembler FRG file from the Illumina reads using fastqToCA (from the Celera Assembler package version 8.3rc2):

```
$ fastqToCA -libraryname illumina -technology illumina -type sanger -innie \
  -insertsize 200 60 -mates reads1.fastq,reads2.fastq > illumina.frg
```

then ran PBcR in this way:

```
$ PBcR -length 500 -partitions 200 -l S288c -s pbcr.spec  -fastq reads.fastq  \
  genomeSize=12160000  illumina.frg
```

For datasets at low read depth ($\leq 31X$) we also requested the following parameters (from http://wgs-assembler.sourceforge.net/wiki/index.php/PBcR#Low_Coverage_Assembly):

```
QV=52
asmOvlErrorRate = 0.1
asmUtgErrorRate = 0.06
asmCgwErrorRate = 0.1
asmCnsErrorRate = 0.1
asmOBT=1
batOptions=-RS -CS
utgGraphErrorRate = 0.05
utgMergeErrorRate = 0.05
asmObtErrorRate=0.08
asmObtErrorLimit=4.5
```

- **Canu.** Like PBcR, Canu is a fork of the Celera assembler. Canu can run without specifying any parameter as it automatically detects the available resources and scale the requirements accordingly. Before assembling the reads, Canu performs a base-error correction step aligning all long reads against each other with MHAP. We used version 1.3 and ran it as:

  ```
  $ canu -p yeast -d yeast genomeSize=12.16m useGrid=0 -nanopore-raw reads.fastq
  ```

  for ONT reads, and as:

  ```
  $ canu -p yeast -d yeast genomeSize=12.16m useGrid=0 -pacbio-raw reads.fastq
  ```

  for PacBio reads. When running on the low coverage ONT and PacBio datasets ($\leq 31X$) we also required:

  ```
  corMhapSensitivity=high corMinCoverage=2 errorRate=0.025 minOverlapLength=499 \
    corMaxEvidenceErate=0.3
  ```

- **SMARTdenovo.** SMARTdenovo aligns the raw ONT or PacBio reads all-vs-all without any error-correction stage using the built-in wtzmo aligner, based on homomer-collapsed seeds. Once the long reads are aligned all against all, a consensus algorithm generates the final assembly. We generated the final consensus running SMARTdenovo 1.0 with default parameters.

- **ABruijn.** ABruijn is the only long-read assembler considered here that is not based on the OLC paradigm, but on a generalized and more flexible De-Bruijn graph approach called A-Bruijn, which can accommodate and assemble error-prone reads. From the generated A-Bruijn graph, an error-prone draft assembly is built, the long reads are aligned against it by BLASR and finally partial order alignment[5] is used to correct the draft assembly. We ran ABruijn 0.4b with default parameters.

We also tested the assembly-polishing tool Nanopolish on a ONT-based Canu assembly. Nanopolish uses the nanopore event current information and run an iterative HMM statistic algorithm to improve the assembly accuracy. We ran Nanopolish 0.5.0 on a Canu assembly, canu.fasta, following the author's recipe:

```
$ bwa mem -x ont2d -t 10 canu.fasta reads.fasta | \
   samtools view -Sb - | samtools sort -f - canu.sorted.bam
$ samtools index canu.sorted.bam
$ cp nanopolish/etc/r9-models/* .
$ nanopolish eventalign -t 10 --sam -r reads.fasta -b canu.sorted.bam -g canu.fasta \
   --models nanopolish_models.fofn | samtools view -Sb - | \
   samtools sort -f - canu.eventalign.sorted.bam
$ samtools index canu.eventalign.sorted.bam
$ python nanopolish/scripts/nanopolish_makerange.py canu.fasta | parallel --results \
  nanopolish.results -P 5 nanopolish variants --consensus polished.{1}.fa -w {1} \
  -r reads.fasta -b canu.sorted.bam -g canu.fasta -e canu.eventalign.sorted.bam \
  -t 10 --min-candidate-frequency 0.1 --models nanopolish_models.fofn
$ python nanopolish/scripts/nanopolish_merge.py polished.*.fa > polished_genome.fa
```

We used three scaffolding pipelines to build scaffolds for an Illumina-only assembly generated by SPAdes v3.7.1, which was run with standard settings plus the `--careful` option. The scaffolding pipelines and the parameters we used to assess them are:

- **HybridSPAdes.** HybridSPAdes is part of the SPAdes genome assembler pipeline. It uses both NGS and long reads to generate an assembly. It first uses the standard SPAdes algorithm to build a De Bruijn graph with the short reads, simplifies it to an assembly graph and then aligns against it the long reads for gap closure. We ran HybridSPAdes using SPAdes v3.7.1 with default parameters plus the `--careful` option.

- **npScarf.** The npScarf pipeline is a scaffolding tool provided within the Japsa package (https://github.com/mdcao/japsa). It takes as input an initial NGS-based draft assembly from SPAdes and a bam alignment file between the long reads and the draft assembly, obtained with BWA[6]. The bam alignments are then used to scaffold contigs and resolve repeat regions. npScarf can make use of the MinION real-time sequencing feature as it can be fed long reads from a stream. We used npScarf from the Japsa package version 1.6-08a with default parameters in its non-real-time fashion. To generate the bam file, we used bwa mem (version 0.7.12) with the following parameters: `-x ont2d -a -Y` for ONT data, and `-x pacbio -a -Y` for PacBio data.

Table S1. Statistic information for the 2D-All and 2D-Pass ONT datasets for the S288C strain.

Oxford Nanopore Datasets							
Strain	Dataset	Bases (Mb)	Reads	Average (b)	Longest (b)	N50 (b)	Identity
S288c	2D, All: 61X	738	90,791	8,123	245,845	11,075	93.3%
	2D, Pass: 31X	383	42,325	9,040	56,477	11,693	93.3%

- **SMIS.** SMIS (https://github.com/fg6/smis.git), or the Single Molecule Integrated Scaffolding pipeline, is in development at the Wellcome Trust Sanger Institute and aims to be a comprehensive pipeline for long reads exploitation, from scaffolding of fragmented NGS-based assemblies to structure variation detection. We assessed the SMIS capabilities as a scaffolding tool. We presented the SMIS scaffolding results when using as input the SPAdes assembly generated as described above and the long reads. From each long read, SMIS creates fake-mates sequences with fixed length and fixed insert length (2000 bp and 200 bp, respectively). Such fake-mates are then aligned against the SPAdes assembly via BWA. If enough fake-mates bridge multiple contigs, the latter are scaffolded together and the gap size is estimated from the initial fixed insert and filled with 'Ns'.

PacBio datasets

The PacBio datasets are available from the EBI database with accession code PRJEB7245. For this study we collected data from the following accession numbers:

- S288C strain: ERR1655125, ERR1655118, and ERR1655119
- SK1 strain : ERR1080537, ERR1080529, ERR1140978 ERR1080522, ERR1080536, and ERR1124245
- N44 strain : ERR1080523, ERR1080530, and ERR1080535
- CBS432 strain : ERR1080527, ERR1080540, and ERR1080541

The appropriate data can be downloaded and merged in fastq files using the scripts available from GitHub: https://github.com/fg6/YeastStrainsStudy.git.

Extraction of the 31X ONT-Emu PacBio subset

We provide a python code to select a subset of reads with desired depth from an initial fasta/fastq file: https://github.com/fg6/random_subreads. The subset can be extracted completely randomly, or following a Gaussian distribution around a desired length position.

The randomly selected subset will have a read length distribution similar to the initial dataset. This is because each read has the same probability to be picked, and there are more reads with length around the initial distribution peak. To modify the distribution shape, for instance to have a peak in a different position, we can modify the probability for a read to be selected depending on its length by assigning a weight: reads with length around the initial distribution peak will have a smaller weight (=smaller probability to be picked), while reads at lengths around the new, desired peak will have higher weight. Then we can select the reads (pseudo-)randomly taking into account the assigned weights. The weight to assign to each read can be tricky to determine and depends on how different the initial and the desired shapes are, but also on how much we want to subset the sample: the larger the final subset, the more difficult it will be to change the original shape.

For the PacBio ONT-Emu datasets, we generated PacBio subsamples with shapes similar to that of the ONT 2D-Pass datasets. For this particular case, we created a new branch of the mentioned repository called "YeastStrainsStudy" that incorporates the heuristically optimized weights to be assigned to each read according to its length. Because of its partially random nature, the subsamples generated contain each time a different group of reads. The exact group of reads used in this study for the 31X ONT-Emu subsample can be obtained using the scripts available from GitHub: https://github.com/fg6/YeastStrainsStudy.git.

Oxford Nanopore: S288c 2D-Pass versus 2D-All data

Here, we compare the *de novo* assemblies from ONT data when using only the best 2D reads, *i.e.* the 2D-Pass reads, to when we use all the 2D reads, *i.e.* including the 2D reads from both the 'Pass' and the 'Fail' directories (2D-All). While the 'Fail' reads' accuracies are lower than those of the Pass reads, they might comprise longer reads which could improve the contiguity

Table S2. Statistic information for the assemblies from the 2D-All and 2D-Pass ONT datasets for the S288C strain.

Oxford Nanopore S288C Datasets												
Dataset	Assembler	Bases (Mb)	Contigs	N50 (kb)	Reference Coverage	SNPs,Indels (# per kb)	Identity	MisAss	Na50 (kb)	Genes (6,615)	CPU Time (h)	Memory (GB)
2D, All 61X	PBcR-MiSeQ	**12.0**	83	324	99.15%	0.2, **0.2**	**99.94%**	**14**	315	6,520	91	16
	Miniasm	11.8	24	760	87.58%	36, 66	88.34%	37	145	2,428	**0.1**	9
	Racon	**12.0**	24	**777**	**99.37%**	0.4, 12	**98.75%**	21	**720**	**6,546**	14	9
	Falcon	11.7	42	711	98.52%	0.5, 26	97.30%	19	705	6,471	57	71
	SMARTdenovo	12.0	**23**	772	99.21%	0.3, 15	98.39%	30	657	6,539	3	**5**
	ABruijn	11.9	24	691	99.18%	0.2, 18	98.15%	30	531	6,531	75	10
	PBcR-Self	15.1	185	408	98.84%	0.2, 24	97.45%	106	290	6,462	310	28
	Canu	11.9	26	650	99.00%	**0.1**, 20	97.94%	34	524	6,462	184	69
2D, Pass 31X	PBcR-MiSeQ	11.9	76	305	99.08%	**0.1, 0.2**	**99.94%**	**18**	273	6,514	147	17
	Miniasm	11.8	27	739	94.85%	34, 67	89.42%	26	362	3,353	**0.1**	**5**
	Racon	12.0	27	752	98.80%	0.4, 11	**98.76%**	24	534	6,533	8	5
	Falcon	11.9	43	717	99.09%	0.5, 21	97.79%	27	**546**	6,526	19	71
	SMARTdenovo	**12.1**	28	625	99.54%	0.3, 14	98.50%	25	531	6,556	2	**5**
	ABruijn	12.4	**26**	**769**	98.89%	**0.1**, 15	98.49%	31	536	6,533	44	8
	PBcR-Self	12.9	64	616	99.21%	0.2, 17	98.24%	92	525	6,552	695	23
	Canu	**12.1**	29	698	**99.62%**	**0.1**, 17	98.30%	34	530	**6,566**	80	14

of the assemblies. The reads statistic information for the 2D-All and 2D-Pass are summarized in the Supplementary Table S1, while the related assemblies' information are shown in the Supplementary Table S2.

The assemblies from the 2D-All or 2D-Pass samples do not differ significantly, but, except for a couple of cases, the assemblies have typically longer reference coverage and slightly higher accuracy when running on the higher quality 2D-Pass dataset; also, most pipelines are able to reconstruct more genes in the 2D-Pass case. The assemblies contiguity though appear higher on the 2D-All sample, as shown by the assembly Na50s. From the resource point of view, the inclusion of the 'Fail' data increased the depth from 31X to 61X, and this resulted in a 2-3 fold longer running time in almost all assemblies except for Miniasm and PBcR. For the latter the running time was about 2 times longer when running on 2D-Pass data, probably because of the additional higher sensitivity parameters used for the lower depth case (see Supplementary Note). When running on the smaller dataset (2D-Pass) the maximum memory requirement slightly decreased or remained the same for all the pipelines except for Miniasm and Canu for which the memory needed was 2 and 4 times lower than on the 2D-All case, respectively.

Even though the Na50s are longer for the 2D-All data, we decided to present our assessment studies using the 2D-Pass based assemblies because of their higher accuracies.

Depth study II: PacBio samples at 120X, 80X, 61X, 31X, 20X and 10X

Statistic information for the assemblies based on the whole S288c PacBio dataset and its randomly selected subsets are shown in the Supplementary Table S5. From 10X to 31X the performances are similar to the one observed for the ONT-Emu samples in Table 4, with PBcR-MiSeQ providing the longest, more accurate assembly at 10X and the other Celera-based pipelines catching up quickly already at 20X although with a lower accuracy. At 31X Canu and PBcR-Self generated the highest accuracies between the non-hybrid pipelines, around 99.9%, second only to the 99.97% accuracy of the hybrid pipeline PBcR-MiSeQ. PBcR-Self produced the most contiguous assembly, with an Na50 of 740 Mb, while SMARTdenovo generated the assembly with the longest reference coverage and the highest number of genes reconstructed, even though with a slightly lower accuracy than Falcon or ABruijn. The accuracy kept slightly improving for all pipelines until 61X depth to remain basically unchanged afterwards, while the Na50 keep increasing until 80X, but did not change or got slightly worse when approaching the depth of 120X. In conclusion, Canu and PBcR-Self were the best performing pipelines for the datasets we analyzed in this study, providing assemblies with high reference coverage, high accuracy and high Na50s already at 31X depth. Increasing the depth beyond 31X improved their accuracy from 99.9% up to 99.97 – 99.98%, a level commonly reached by Illumina-only assemblies. Unlike the Illumina-only assemblies, they achieved quite long Na50s: 549 kb for Canu and 740 kb for PBcR-Self.

Also Falcon and SMARTdenovo reached long Na50s (at 120X depth 740 kb and 667 kb, respectively), but their accuracies remained at a lower 99.9%; a similar value for the accuracy was provided by ABruijn, which produced slightly lower Na50 (546 kb at 120X) but was able to reconstruct more genes. The highest number of genes (6,608 out of 6,615) was reconstructed by Canu when run on the 80X and the 120X samples.

Other Strains Assemblies from PacBio Data

For the N44, CBS432, and SK1 strains no reference genome exists. We *de novo* assembled the 148X depth N44, the 135X depth CBS432, and the 248X depth SK1 PacBio data with the same assembler pipelines used for the S288C strain to obtain very contiguous assemblies whose statistic information are summarized in the Supplementary Table S4. While we cannot directly estimate the assembly accuracies, contiguity and possible misassemblies, we can expect these assemblies to have similar accuracy obtained for the 120X depth S288c PacBio data, up to 99.98% for the Celera-based assemblers. Table S4 also shows that Canu is the pipeline that reconstruct for each strain the highest number of genes. It also suggests that the SK1 strain has an higher number of genes in common with the reference strain than CBS432 and N44, as expected. A comprehensive structure-variation analysis of these strains using the same PacBio datasets used here can be found in[7].

References

1. Broder, A. Z. On the resemblance and containment of documents. In *In Compression and Complexity of Sequences (SEQUENCES'97)*, 21–29 (IEEE Computer Society, 1997).

2. Sović, I. *et al.* Fast and sensitive mapping of nanopore sequencing reads with GraphMap. *Nature Communications* **7, 11307** (2016).

3. Miller, J. R. *et al.* Aggressive assembly of pyrosequencing reads with mates. *Bioinformatics* **24**, 2818–2824 (2000).

4. Berlin, K. *et al.* Assembling large genomes with single-molecule sequencing and locality sensitive hashing. *Nature Biotechnology* **33**, 623–630 (2015).

5. Lee, G. C. S. M., C. Multiple sequence alignment using partial order graphs. *Bioinformatics* **18**, 452–464.

6. Li, H. Aligning sequence reads, clone sequences and assembly contigs with bwa-mem. *arXiv:* 1303.3997v1 [q–bio.GN] (2013).

7. Yue, J.-X. *et al.* Contrasting genome dynamics between domesticated and wild yeasts. bioRxiv; 10.1101/076562 (2016).

Table S3. Statistic information for the *de novo* assemblies generating using, from top to bottom: the whole S288c PacBio dataset, subsets at 80X, 61X, 31X, 20X and 10X depth of randomly selected reads from the immediately larger subset.

Pacific Biosciences S288C Datasets												
Dataset	Assembler	Bases (Mb)	Contigs	N50 (kb)	Reference Coverage	SNPs,Indels (# per kb)	Identity	MisAss	Na50 (kb)	Genes (6,615)	CPU Time (h)	Memory (GB)
Whole: 120X	PBcR-MiSeQ	11.8	70	379	98.78%	0.1, **0.1**	99.96%	**15**	315	6,532	172	18
	Miniasm	12.5	29	669	94.72%	20, 88	89.23%	64	109	3,075	**0.4**	15
	Racon	12.0	29	642	98.59%	0.2, 2	99.77%	27	536	6,528	65	15
	Falcon	11.9	24	805	98.45%	0.2, 1	99.90%	18	**740**	6,515	82	75
	SMARTdenovo	12.1	18	778	99.16%	0.1, 1	99.93%	30	667	6,581	5	**5**
	ABruijn	12.3	20	776	99.88%	**0.04**, 1	99.90%	45	546	6,604	42	19
	PBcR-Self	**12.2**	**17**	**810**	99.27%	0.1, 0.2	99.97%	34	**740**	6,588	29	32
	Canu	12.3	22	778	**99.95%**	0.1, **0.1**	**99.98%**	34	549	**6,606**	35	15
Random: 80X	PBcR-MiSeQ	11.8	62	421	98.92%	**0.1, 0.1**	99.96%	**12**	314	6,538	172	19
	Miniasm	12.5	26	810	94.96%	19, 87	89.27%	78	88	3,135	**0.3**	13
	Racon	12.0	26	777	98.99%	0.2, 2	99.75%	25	634	6,539	49	13
	Falcon	11.9	25	809	98.75%	0.2, 1	99.91%	15	726	6,541	124	75
	SMARTdenovo	12.1	**19**	779	99.11%	**0.1**, 1	99.90%	27	667	6,581	4	**5**
	ABruijn	**12.2**	22	737	99.91%	**0.1**, 1	99.90%	40	548	6,605	29	12
	PBcR-Self	12.5	36	777	99.35%	**0.1**, 0.2	99.96%	54	637	6,588	21	32
	Canu	12.3	20	**814**	**99.97%**	**0.1, 0.1**	**99.98%**	39	**740**	**6,608**	29	14
Random: 61X	PBcR-MiSeQ	11.9	72	425	99.46%	**0.1, 0.1**	**99.97%**	**12**	315	6,558	98	19
	Miniasm	12.6	31	586	95.38%	19, 87	89.38%	69	111	3,213	**0.2**	9
	Racon	**12.1**	31	567	99.34%	**0.1**, 2	99.73%	27	540	6,564	38	9
	Falcon	12.0	25	766	99.03%	0.2, 1	99.89%	23	661	6,570	61	73
	SMARTdenovo	12.3	23	744	98.54%	**0.1**, 1	99.85%	27	547	**6,602**	3	**4**
	ABruijn	**12.1**	**20**	**818**	99.60%	**0.1**, 1	99.90%	26	**740**	6,592	19	10
	PBcR-Self	12.3	29	743	99.32%	**0.1**, 0.3	99.96%	32	568	6,588	16	30
	Canu	12.3	22	778	99.93%	**0.1**, 0.2	**99.97%**	29	565	6,601	23	14
Random: 31X	PBcR-MiSeQ	11.7	76	272	98.05%	**0.1, 0.1**	**99.97%**	**12**	266	6,444	129	17
	Miniasm	12.5	46	455	95.59%	19, 87	89.43%	75	76	3,300	**0.01**	5
	Racon	12.0	46	443	98.92%	0.2, 4	99.51%	26	423	6,530	18	5
	Falcon	12.0	45	501	98.13%	0.3, 2	99.78%	32	447	6,487	33	64
	SMARTdenovo	**12.3**	**30**	556	**99.93%**	0.2, 4	99.53%	25	510	**6,588**	2	**4**
	ABruijn	11.9	58	273	97.81%	**0.1**, 1	99.81%	29	245	6,476	22	7
	PBcR-Self	12.4	38	**813**	99.23%	0.2, 1	99.91%	34	**740**	6,580	69	26
	Canu	**12.3**	37	530	99.70%	**0.1**, 0.5	99.92%	30	496	6,580	21	10
Random: 20X	PBcR-MiSeQ	11.7	72	263	97.99%	**0.1, 0.1**	**99.97%**	**14**	263	6,481	85	13
	Miniasm	11.2	167	89	87.48%	16, 77	89.61%	42	36	3,076	**0.04**	**3**
	Racon	10.8	167	85	90.08%	0.5, 7	99.14%	21	82	5,824	12	2
	Falcon	9.8	210	80	82.32%	0.3, 2	99.66%	31	76	5,280	11	41
	SMARTdenovo	12.1	61	307	98.63%	1, 11	98.77%	23	265	6,402	1	**3**
	ABruijn	9.6	102	106	80.83%	0.2, 3	99.53%	20	102	5,273	20	9
	PBcR-Self	12.5	61	**570**	99.0%	0.2, 2	99.75%	45	**435**	6,557	35	21
	Canu	**12.2**	**42**	438	**99.60%**	0.2, 2	99.80%	25	321	**6,570**	10	7
Random: 10X	PBcR-MiSeQ	**10.7**	**189**	**91**	**91.86%**	**0.1, 0.1**	**99.96%**	**11**	**87**	**5,926**	**46**	**7**
	Miniasm	2.1	87	26	19.34%	3, 14	89.65%	4	15	580	0.02	0.1
	Racon	2	87	25	20.27%	0.4, 2	98.19%	8	23	1,107	6	1
	Falcon	0.7	96	11	10.34%	0.1, 0.3	99.24%	8	10	484	2	23
	SMARTdenovo	6.8	174	42	57.87%	3, 20	95.77%	10	37	3,284	0.4	1
	ABruijn	1.4	22	67	15.76%	0.2, 2	98.34%	6	67	877	5	7
	PBcR-Self	8.5	264	41	70.17%	0.6, 7	98.88%	34	40	4,389	12	19
	Canu	8.0	207	44.6	69.11%	0.4, 5	99.13%	9	44	4,348	4	7

Table S4. Statistic information for the PacBio *de novo* assemblies from the PBcR-MiSeQ, Miniasm, Racon, Falcon, SMARTdenovo, ABruijn, PBcR-Self and Canu pipelines for the N44 (top panel), CBS432 (middle panel), and SK1 (bottom panel) strains.

Pacific Biosciences Datasets							
Dataset	Assembler	Bases (Mb)	Contigs	N50 (kb)	Genes (6,615)	CPU Time (h)	Memory (GB)
N44	PBcR-MiSeQ	12.0	57	554	5,531	138	18
	Miniasm	12.1	17	830	247	1	16
	Racon	11.7	17	801	5,472	101	17
	Falcon	11.7	22	789	5,507	92	73
	SMARTdenovo	11.9	18	801	5,540	4	4
	ABruijn	12.0	19	797	5,550	117	20
	PBcR-Self	11.9	22	800	5,544	49	30
	Canu	11.9	18	800	5,552	46	14
CBS432	PBcR-MiSeQ	12.0	68	395	5,464	218	19
	Miniasm	12.4	20	853	301	1	16
	Racon	11.9	20	826	5,549	72	16
	Falcon	11.8	24	809	5,573	90	72
	SMARTdenovo	12.0	20	827	5,598	4	4
	ABruijn	12.1	19	779	5,608	59	18
	PBcR-Self	12.2	33	816	5,601	39	31
	Canu	12.2	25	742	5,609	41	14
SK1	PBcR-MiSeQ	11.8	59	413	6,369	110	17
	Miniasm	12.3	22	840	3,587	2	19
	Racon	12.0	22	814	6,467	137	29
	Falcon	10.4	104	272	5,697	218	80
	SMARTdenovo	12.1	19	819	6,490	10	5
	ABruijn	12.1	18	822	6,502	241	37
	PBcR-Self	12.4	33	829	6,490	68	30
	Canu	12.3	24	830	6,504	98	14

Table S5. Chromosome Reconstruction. Chromosome reconstruction percentage and number of contigs for the S288C ONT 2D-Pass 31X dataset as estimated using Quast output. The mitochondrial genome is labeled as 'mt'.

Oxford Nanopore 2D-Pass (31X) Dataset								
Chr	PBcR-MiSeq %, Contigs	Miniasm %, Contigs	Racon %, Contigs	Falcon %, Contigs	SMARTdenovo %, Contigs	ABruijn %, Contigs	PBcR-Self %, Contigs	Canu %, Contigs
I	98.5%, 5	72.5%, 1	86.2%, 1	88.9%, 1	91.0%, 1	88.9%, 1	97.1%, 2	92.7%, 1
II	98.1%, 4	88.3%, 1	100.0%, 1	98.8%, 1	99.1%, 1	96.7%, 2	99.7%, 2	100.0%, 1
III	97.8%, 5	81.2%, 2	90.5%, 2	95.3%, 3	95.7%, 2	90.6%, 2	98.7%, 2	98.0%, 2
IV	96.9%, 9	86.0%, 5	99.6%, 5	97.6%, 3	98.5%, 4	99.4%, 4	100.0%, 7	99.9%, 3
V	97.8%, 3	78.9%, 2	99.5%, 2	97.3%, 2	100.0%, 2	98.7%, 2	100.0%, 5	100.0%, 2
VI	94.4%, 2	86.6%, 1	100.0%, 1	89.2%, 2	100.0%, 1	98.1%, 1	99.8%, 1	100.0%, 1
VII	98.1%, 8	86.8%, 2	100.0%, 4	98.1%, 3	100.0%, 2	98.3%, 3	100.0%, 4	100.0%, 5
VIII	96.2%, 3	82.4%, 4	96.6%, 2	98.6%, 3	95.8%, 3	98.6%, 1	99.9%, 3	97.5%, 2
IX	98.9%, 2	91.2%, 1	99.6%, 2	94.2%, 1	97.0%, 2	95.5%, 1	99.8%, 3	97.2%, 1
X	97.3%, 5	83.3%, 2	97.3%, 3	98.1%, 2	97.6%, 4	98.8%, 2	97.9%, 5	98.8%, 4
XI	100.0%, 1	87.6%, 1	99.4%, 1	97.6%, 1	100.0%, 1	98.9%, 1	99.9%, 1	100.0%, 1
XII	95.3%, 10	84.0%, 6	95.9%, 4	98.3%, 11	99.5%, 5	97.8%, 8	99.0%, 40	98.9%, 6
XIII	98.3%, 6	92.4%, 5	100.0%, 4	98.3%, 3	99.8%, 2	99.6%, 2	99.9%, 4	100.0%, 2
XIV	98.6%, 7	89.1%, 1	100.0%, 1	97.7%, 5	99.1%, 1	99.1%, 1	99.9%, 4	99.8%, 1
XV	88.3%, 6	87.4%, 2	100.0%, 1	97.0%, 1	99.9%, 2	99.3%, 2	100.0%, 4	100.0%, 2
XVI	97.2%, 9	87.7%, 3	99.7%, 3	98.8%, 6	99.9%, 2	99.4%, 2	98.7%, 3	99.9%, 2
mt	96.3%, 2	0.0%, 0	0.0%, 0	66.7%, 4	0.0%, 0	23.7%, 1	0.0%, 0	63.7%, 2

Table S6. Chromosome Reconstruction. Chromosome reconstruction percentage and number of contigs for the S288C PacBio ONT-Emu 31X subset as estimated using Quast output. The mitochondrial genome is labeled as 'mt'.

PacBio ONT-Emu 31X Dataset								
Chr	PBcR-MiSeq %, Contigs	Miniasm %, Contigs	Racon %, Contigs	Falcon %, Contigs	SMARTdenovo %, Contigs	ABruijn %, Contigs	PBcR-Self %, Contigs	Canu %, Contigs
I	96.6%, 3	58.0%, 1	89.7%, 1	88.2%, 3	89.3%, 1	91.2%, 1	89.7%, 2	95.4%, 2
II	98.5%, 4	69.4%, 2	100.0%, 2	94.6%, 4	100.0%, 1	99.5%, 1	99.7%, 1	100.0%, 1
III	96.8%, 4	72.4%, 2	91.0%, 2	98.4%, 1	99.0%, 2	96.3%, 2	98.9%, 2	99.0%, 1
IV	97.1%, 10	74.1%, 7	99.2%, 7	99.0%, 7	100.0%, 3	99.8%, 7	100.0%, 4	99.1%, 5
V	97.6%, 4	68.4%, 2	96.3%, 1	96.3%, 2	100.0%, 1	100.0%, 1	99.7%, 1	100.0%, 1
VI	99.2%, 2	70.1%, 1	100.0%, 1	96.0%, 1	100.0%, 1	99.3%, 1	100.0%, 2	100.0%, 1
VII	97.9%, 9	67.0%, 3	98.3%, 2	98.9%, 2	100.0%, 2	99.9%, 2	100.0%, 2	100.0%, 2
VIII	96.7%, 2	63.9%, 2	97.9%, 2	95.9%, 3	98.8%, 3	95.8%, 3	100.0%, 3	100.0%, 1
IX	98.8%, 1	54.4%, 1	97.0%, 1	97.2%, 1	95.2%, 1	99.0%, 1	99.4%, 1	97.6%, 1
X	96.0%, 4	80.1%, 2	98.7%, 3	98.4%, 3	98.7%, 2	96.9%, 3	99.9%, 2	98.1%, 2
XI	100.0%, 1	66.0%, 1	99.7%, 1	96.9%, 1	100.0%, 1	99.9%, 1	100.0%, 1	100.0%, 1
XII	94.3%, 10	60.3%, 3	95.7%, 4	94.6%, 4	98.8%, 3	98.6%, 6	99.1%, 19	99.1%, 7
XIII	98.3%, 8	63.1%, 4	98.4%, 5	96.8%, 4	99.5%, 2	99.4%, 3	100.0%, 3	100.0%, 3
XIV	98.1%, 8	65.4%, 2	96.4%, 2	98.5%, 4	99.2%, 1	99.0%, 1	99.1%, 3	99.1%, 1
XV	98.7%, 5	70.2%, 2	98.0%, 3	97.7%, 4	99.6%, 2	98.7%, 3	99.9%, 4	100.0%, 3
XVI	96.6%, 8	59.9%, 3	97.4%, 3	98.6%, 3	100.0%, 1	99.8%, 3	98.7%, 1	99.5%, 3
mt	0.0%, 0	77.0%, 1	100.0%, 1	42.8%, 3	100.0%, 1	42.0%, 1	31.0%, 1	100.0%, 1
